# 2-(2-Methyl-5-nitro-1*H*-imidazol-1-yl)ethyl *N*-methyl­carbamate

**DOI:** 10.1107/S1600536808041676

**Published:** 2008-12-13

**Authors:** Gui-Yun Duan, Cheng-Cai Xia, Yu-Liang Xiao

**Affiliations:** a College of Pharmaceutical Sciences, Taishan Medical University, Tai’an 271016, People’s Republic of China

## Abstract

In the title compound, C_8_H_12_N_4_O_4_, the essentially planar methyl­carbamoyloxymethyl group [maximum deviation 0.038 (3) Å] and the imidazole ring make a dihedral angle of 48.47 (3)°. The crystal packing is stabilized by inter­molecular N—H⋯N and C—H⋯O hydrogen bonds, which link the mol­ecules into infinite ribbons running along the *a* axis, and by weak π–π stacking inter­actions [centroid–centroid distance = 3.894 (2) Å].

## Related literature

For biological activity, see: Cina *et al.* (1996 ▶); Karamanakos *et al.* (2007 ▶). For bond-length data, see: Allen *et al.* (1987 ▶).
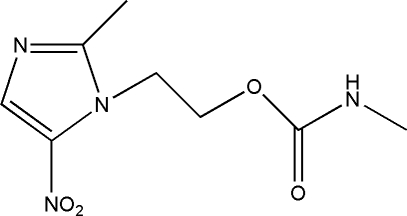


## Experimental

### 

#### Crystal data

C_8_H_12_N_4_O_4_
*M* *_r_* = 228.22
Monoclinic, 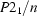

*a* = 9.6959 (12) Å
*b* = 7.2898 (9) Å
*c* = 15.589 (2) Å
β = 101.400 (2)°
*V* = 1080.1 (2) Å^3^
*Z* = 4
Mo *K*α radiation
μ = 0.11 mm^−1^
*T* = 273 (2) K
0.15 × 0.12 × 0.10 mm

#### Data collection

Bruker SMART CCD area-detector diffractometer
Absorption correction: none
5515 measured reflections
1921 independent reflections
1622 reflections with *I* > 2σ(*I*)
*R*_int_ = 0.015

#### Refinement

*R*[*F* ^2^ > 2σ(*F* ^2^)] = 0.031
*wR*(*F* ^2^) = 0.089
*S* = 1.05
1921 reflections
146 parameters
H-atom parameters constrained
Δρ_max_ = 0.19 e Å^−3^
Δρ_min_ = −0.14 e Å^−3^

### 

Data collection: *SMART* (Bruker, 2001 ▶); cell refinement: *SAINT* (Bruker, 2001 ▶); data reduction: *SAINT*; program(s) used to solve structure: *SHELXTL* (Sheldrick, 2008 ▶); program(s) used to refine structure: *SHELXTL*; molecular graphics: *SHELXTL*; software used to prepare material for publication: *SHELXTL*.

## Supplementary Material

Crystal structure: contains datablocks I, global. DOI: 10.1107/S1600536808041676/hg2452sup1.cif

Structure factors: contains datablocks I. DOI: 10.1107/S1600536808041676/hg2452Isup2.hkl

Additional supplementary materials:  crystallographic information; 3D view; checkCIF report

## Figures and Tables

**Table 1:** Hydrogen-bond geometry (Å, °)

*D*—H⋯*A*	*D*—H	H⋯*A*	*D*⋯*A*	*D*—H⋯*A*
N4—H4⋯N1^i^	0.86	2.20	3.0416 (17)	165
C2—H2⋯O4^ii^	0.93	2.34	3.2492 (18)	166
C8—H8*B*⋯O4^iii^	0.96	2.57	3.498 (2)	164

Symmetry codes: (i) 
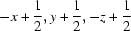
; (ii) 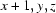; (iii) 
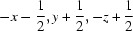
.

**Fig. 1.**
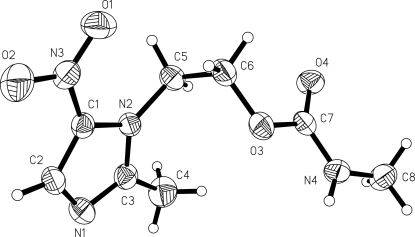
View of the title compound (I), with displacement ellipsoids drawn at the 40% probability level.

## supplementary crystallographic information

### Comment

2-(2-methyl-5-nitro-1*H*-imidazol-1-yl)ethanol is an anti-anaerobic bacteria and anti-infusorium agent to treatment of infection which is called metronidazole (Cina *et al.*, 1996; Karamanakos *et al.*, 2007). The title compound is a derivative of it. In this paper, we report the crystal structure of the title compound.

In (I) (Fig. 1), all bond lengths are normal (Allen *et al.*, 1987). Atoms C4, C5, N3, O1, O2 lie in the plane of the imidazole ring (C1/C2/C3/N1/N2) with maximum deviations 0.004 (2)Å for O1. The essentially planar methyl methylcarbamate moiety (C6—C8/N4/O3/O4) and the imidazole ring make a dihedral angle of 48.47 (3)°. The relatively short distance of 3.894 (2)Å between the centroids of imidazole ring C1/C2/C3/N1/N2 [at (1-*X*,-Y,1-*Z*)] indicates the presence of weak π-π interactions, which contribute to the stability of the crystal packing. The crystal packing is also stabilized by intermolecular N—H···N and C—H···O hydrogen bonds,

### Experimental

The title compound was prepared by reaction of 1.71 g (0.01 mol) 2-(2-methyl-5-nitro-1*H*-imidazol-1-yl)ethanol with 0.68 g (0.12 mol) methyl isocyanate catalyzed by 1 g triethylamine in 20 ml toluene at room temperature, yield 86.4%. Crystals suitable for X-ray diffraction analysis were obtained by slow evaporation of a metanol solution at room temperature for two weeks.

### Refinement

All H atoms were placed in calculated positions, with C—H = 0.93–0.97 Å, N—H = 0.86 Å, and included in the final cycles of refinement using a riding model, with *U*_iso_(H) = 1.2 (1.5 for methyl) times *U*_eq_(C, N).

### Crystal data

**Table:**

C_8_H_12_N_4_O_4_	*F*(000) = 480
*M**_r_* = 228.22	*D*_x_ = 1.403 Mg m^−^^3^
Monoclinic, *P*2_1_/*n*	Mo *K*α radiation, λ = 0.71073 Å
Hall symbol: -P 2yn	Cell parameters from 2505 reflections
*a* = 9.6959 (12) Å	θ = 2.7–27.1°
*b* = 7.2898 (9) Å	µ = 0.11 mm^−^^1^
*c* = 15.589 (2) Å	*T* = 273 K
β = 101.400 (2)°	Block, yellow
*V* = 1080.1 (2) Å^3^	0.15 × 0.12 × 0.10 mm
*Z* = 4	

### Data collection

**Table:**

Bruker SMART CCD area-detector diffractometer	1622 reflections with *I* > 2σ(*I*)
Radiation source: fine-focus sealed tube	*R*_int_ = 0.015
graphite	θ_max_ = 25.0°, θ_min_ = 2.3°
φ and ω scans	*h* = −11→11
5515 measured reflections	*k* = −8→8
1921 independent reflections	*l* = −9→18

### Refinement

**Table:**

Refinement on *F*^2^	Secondary atom site location: difference Fourier map
Least-squares matrix: full	Hydrogen site location: inferred from neighbouring sites
*R*[*F*^2^ > 2σ(*F*^2^)] = 0.031	H-atom parameters constrained
*wR*(*F*^2^) = 0.089	*w* = 1/[σ^2^(*F*_o_^2^) + (0.0404*P*)^2^ + 0.2462*P*] where *P* = (*F*_o_^2^ + 2*F*_c_^2^)/3
*S* = 1.05	(Δ/σ)_max_ < 0.001
1921 reflections	Δρ_max_ = 0.19 e Å^−^^3^
146 parameters	Δρ_min_ = −0.14 e Å^−^^3^
0 restraints	Extinction correction: *SHELXTL* (Sheldrick, 2008), Fc^*^=kFc[1+0.001xFc^2^λ^3^/sin(2θ)]^-1/4^
Primary atom site location: structure-invariant direct methods	Extinction coefficient: 0.011 (2)

### Special details

**Table:**

Geometry. All e.s.d.'s (except the e.s.d. in the dihedral angle between two l.s. planes) are estimated using the full covariance matrix. The cell e.s.d.'s are taken into account individually in the estimation of e.s.d.'s in distances, angles and torsion angles; correlations between e.s.d.'s in cell parameters are only used when they are defined by crystal symmetry. An approximate (isotropic) treatment of cell e.s.d.'s is used for estimating e.s.d.'s involving l.s. planes.
Refinement. Refinement of *F*^2^ against ALL reflections. The weighted *R*-factor *wR* and goodness of fit *S* are based on *F*^2^, conventional *R*-factors *R* are based on *F*, with *F* set to zero for negative *F*^2^. The threshold expression of *F*^2^ > σ(*F*^2^) is used only for calculating *R*-factors(gt) *etc*. and is not relevant to the choice of reflections for refinement. *R*-factors based on *F*^2^ are statistically about twice as large as those based on *F*, and *R*- factors based on ALL data will be even larger.

### Fractional atomic coordinates and isotropic or equivalent isotropic displacement parameters (Å^2^)

**Table:**

	*x*	*y*	*z*	*U*_iso_*/*U*_eq_	
O1	0.41899 (13)	0.31179 (19)	0.60227 (7)	0.0673 (4)	
O2	0.62309 (12)	0.35345 (19)	0.56977 (8)	0.0693 (4)	
O3	0.06913 (10)	0.38773 (15)	0.39251 (7)	0.0495 (3)	
O4	−0.13394 (10)	0.24805 (16)	0.40569 (7)	0.0516 (3)	
N1	0.45446 (13)	0.11166 (19)	0.33518 (8)	0.0511 (3)	
N2	0.32310 (11)	0.14897 (15)	0.43564 (7)	0.0368 (3)	
N3	0.50075 (13)	0.29867 (17)	0.55195 (8)	0.0461 (3)	
N4	−0.11627 (13)	0.41147 (18)	0.28483 (8)	0.0489 (3)	
H4	−0.0602	0.4739	0.2598	0.059*	
C1	0.45604 (14)	0.21818 (18)	0.46825 (9)	0.0374 (3)	
C2	0.53355 (15)	0.1938 (2)	0.40604 (10)	0.0456 (4)	
H2	0.6272	0.2283	0.4111	0.055*	
C3	0.32821 (15)	0.0863 (2)	0.35445 (9)	0.0437 (4)	
C4	0.20850 (19)	−0.0011 (3)	0.29424 (12)	0.0657 (5)	
H4A	0.2356	−0.0287	0.2397	0.099*	
H4B	0.1297	0.0812	0.2841	0.099*	
H4C	0.1828	−0.1125	0.3200	0.099*	
C5	0.20058 (15)	0.1428 (2)	0.47777 (10)	0.0453 (4)	
H5A	0.2301	0.0991	0.5373	0.054*	
H5B	0.1326	0.0562	0.4467	0.054*	
C6	0.13134 (15)	0.3268 (2)	0.47920 (10)	0.0489 (4)	
H6A	0.0591	0.3191	0.5141	0.059*	
H6B	0.2007	0.4159	0.5064	0.059*	
C7	−0.06789 (14)	0.34081 (19)	0.36319 (9)	0.0390 (3)	
C8	−0.26021 (17)	0.3868 (2)	0.24023 (11)	0.0564 (4)	
H8A	−0.2734	0.2632	0.2190	0.085*	
H8B	−0.2811	0.4705	0.1918	0.085*	
H8C	−0.3219	0.4104	0.2801	0.085*	

### Atomic displacement parameters (Å^2^)

**Table:**

	*U*^11^	*U*^22^	*U*^33^	*U*^12^	*U*^13^	*U*^23^
O1	0.0660 (8)	0.0934 (10)	0.0442 (6)	−0.0052 (7)	0.0153 (6)	−0.0090 (6)
O2	0.0506 (7)	0.0844 (9)	0.0671 (8)	−0.0186 (6)	−0.0026 (6)	−0.0062 (7)
O3	0.0351 (5)	0.0590 (7)	0.0568 (7)	−0.0004 (5)	0.0146 (5)	0.0064 (5)
O4	0.0431 (6)	0.0645 (7)	0.0486 (6)	−0.0100 (5)	0.0123 (5)	0.0118 (5)
N1	0.0494 (7)	0.0567 (8)	0.0504 (8)	0.0087 (6)	0.0176 (6)	−0.0046 (6)
N2	0.0346 (6)	0.0373 (6)	0.0394 (6)	0.0021 (5)	0.0091 (5)	0.0027 (5)
N3	0.0447 (7)	0.0477 (7)	0.0436 (7)	−0.0008 (6)	0.0029 (6)	0.0050 (6)
N4	0.0441 (7)	0.0564 (8)	0.0491 (7)	−0.0010 (6)	0.0162 (6)	0.0130 (6)
C1	0.0350 (7)	0.0356 (7)	0.0410 (7)	0.0018 (6)	0.0062 (6)	0.0040 (6)
C2	0.0364 (8)	0.0477 (8)	0.0547 (9)	0.0043 (6)	0.0141 (7)	0.0042 (7)
C3	0.0459 (8)	0.0405 (8)	0.0439 (8)	0.0073 (6)	0.0071 (6)	−0.0020 (6)
C4	0.0633 (11)	0.0710 (12)	0.0582 (10)	−0.0002 (9)	0.0007 (8)	−0.0172 (9)
C5	0.0378 (7)	0.0522 (9)	0.0481 (8)	−0.0052 (6)	0.0138 (6)	0.0045 (7)
C6	0.0361 (8)	0.0641 (10)	0.0478 (9)	0.0024 (7)	0.0112 (6)	−0.0061 (7)
C7	0.0359 (7)	0.0385 (7)	0.0460 (8)	0.0023 (6)	0.0162 (6)	−0.0017 (6)
C8	0.0554 (10)	0.0609 (10)	0.0512 (9)	−0.0011 (8)	0.0062 (7)	0.0107 (8)

### Geometric parameters (Å, °)

**Table:**

O1—N3	1.2241 (16)	C1—C2	1.3509 (19)
O2—N3	1.2303 (16)	C2—H2	0.9300
O3—C7	1.3605 (17)	C3—C4	1.485 (2)
O3—C6	1.4370 (18)	C4—H4A	0.9600
O4—C7	1.2140 (16)	C4—H4B	0.9600
N1—C3	1.3297 (19)	C4—H4C	0.9600
N1—C2	1.354 (2)	C5—C6	1.503 (2)
N2—C3	1.3555 (18)	C5—H5A	0.9700
N2—C1	1.3840 (17)	C5—H5B	0.9700
N2—C5	1.4673 (17)	C6—H6A	0.9700
N3—C1	1.4181 (18)	C6—H6B	0.9700
N4—C7	1.3233 (19)	C8—H8A	0.9600
N4—C8	1.442 (2)	C8—H8B	0.9600
N4—H4	0.8600	C8—H8C	0.9600
			
C7—O3—C6	116.02 (11)	H4A—C4—H4C	109.5
C3—N1—C2	105.99 (12)	H4B—C4—H4C	109.5
C3—N2—C1	105.22 (11)	N2—C5—C6	112.52 (12)
C3—N2—C5	126.14 (12)	N2—C5—H5A	109.1
C1—N2—C5	128.64 (11)	C6—C5—H5A	109.1
O1—N3—O2	123.20 (13)	N2—C5—H5B	109.1
O1—N3—C1	120.20 (12)	C6—C5—H5B	109.1
O2—N3—C1	116.60 (13)	H5A—C5—H5B	107.8
C7—N4—C8	121.95 (12)	O3—C6—C5	111.56 (12)
C7—N4—H4	119.0	O3—C6—H6A	109.3
C8—N4—H4	119.0	C5—C6—H6A	109.3
C2—C1—N2	107.35 (12)	O3—C6—H6B	109.3
C2—C1—N3	127.06 (13)	C5—C6—H6B	109.3
N2—C1—N3	125.59 (12)	H6A—C6—H6B	108.0
C1—C2—N1	109.76 (13)	O4—C7—N4	126.27 (13)
C1—C2—H2	125.1	O4—C7—O3	122.83 (13)
N1—C2—H2	125.1	N4—C7—O3	110.89 (12)
N1—C3—N2	111.67 (13)	N4—C8—H8A	109.5
N1—C3—C4	123.81 (14)	N4—C8—H8B	109.5
N2—C3—C4	124.52 (14)	H8A—C8—H8B	109.5
C3—C4—H4A	109.5	N4—C8—H8C	109.5
C3—C4—H4B	109.5	H8A—C8—H8C	109.5
H4A—C4—H4B	109.5	H8B—C8—H8C	109.5
C3—C4—H4C	109.5		
			
C3—N2—C1—C2	−0.11 (15)	C1—N2—C3—N1	0.19 (16)
C5—N2—C1—C2	−179.97 (13)	C5—N2—C3—N1	−179.95 (13)
C3—N2—C1—N3	−179.87 (13)	C1—N2—C3—C4	179.78 (15)
C5—N2—C1—N3	0.3 (2)	C5—N2—C3—C4	−0.4 (2)
O1—N3—C1—C2	−179.83 (15)	C3—N2—C5—C6	−104.57 (16)
O2—N3—C1—C2	0.5 (2)	C1—N2—C5—C6	75.25 (18)
O1—N3—C1—N2	−0.1 (2)	C7—O3—C6—C5	91.29 (14)
O2—N3—C1—N2	−179.77 (13)	N2—C5—C6—O3	66.34 (16)
N2—C1—C2—N1	0.01 (16)	C8—N4—C7—O4	1.1 (2)
N3—C1—C2—N1	179.76 (13)	C8—N4—C7—O3	−178.06 (13)
C3—N1—C2—C1	0.11 (17)	C6—O3—C7—O4	−2.3 (2)
C2—N1—C3—N2	−0.19 (17)	C6—O3—C7—N4	176.83 (12)
C2—N1—C3—C4	−179.78 (15)		

### Hydrogen-bond geometry (Å, °)

**Table:**

*D*—H···*A*	*D*—H	H···*A*	*D*···*A*	*D*—H···*A*
N4—H4···N1^i^	0.86	2.20	3.0416 (17)	165
C2—H2···O4^ii^	0.93	2.34	3.2492 (18)	166
C8—H8B···O4^iii^	0.96	2.57	3.498 (2)	164

Symmetry codes: (i) −*x*+1/2, *y*+1/2, −*z*+1/2; (ii) *x*+1, *y*, *z*; (iii) −*x*−1/2, *y*+1/2, −*z*+1/2.
